# Temperature variability analysis using wavelets and multiscale entropy in patients with systemic inflammatory response syndrome, sepsis, and septic shock

**DOI:** 10.1186/cc11255

**Published:** 2012-03-18

**Authors:** Vasilios E Papaioannou, Ioanna G Chouvarda, Nikos K Maglaveras, Ioannis A Pneumatikos

**Affiliations:** 1 Alexandroupolis University Hospital, Intensive Care Unit, Democritus University of Thrace, Dragana 68100, Greece; 2 Laboratory of Medical Informatics, School of Medicine, Aristotle University of Thessaloniki, 54124, Greece

## Abstract

**Background:**

Even though temperature is a continuous quantitative variable, its measurement has been considered a snapshot of a process, indicating whether a patient is febrile or afebrile. Recently, other diagnostic techniques have been proposed for the association between different properties of the temperature curve with severity of illness in the Intensive Care Unit (ICU), based on complexity analysis of continuously monitored body temperature. In this study, we tried to assess temperature complexity in patients with systemic inflammation during a suspected ICU-acquired infection, by using wavelets transformation and multiscale entropy of temperature signals, in a cohort of mixed critically ill patients.

**Methods:**

Twenty-two patients were enrolled in the study. In five, systemic inflammatory response syndrome (SIRS, group 1) developed, 10 had sepsis (group 2), and seven had septic shock (group 3). All temperature curves were studied during the first 24 hours of an inflammatory state. A wavelet transformation was applied, decomposing the signal in different frequency components (scales) that have been found to reflect neurogenic and metabolic inputs on temperature oscillations. Wavelet energy and entropy per different scales associated with complexity in specific frequency bands and multiscale entropy of the whole signal were calculated. Moreover, a clustering technique and a linear discriminant analysis (LDA) were applied for permitting pattern recognition in data sets and assessing diagnostic accuracy of different wavelet features among the three classes of patients.

**Results:**

Statistically significant differences were found in wavelet entropy between patients with SIRS and groups 2 and 3, and in specific ultradian bands between SIRS and group 3, with decreased entropy in sepsis. Cluster analysis using wavelet features in specific bands revealed concrete clusters closely related with the groups in focus. LDA after wrapper-based feature selection was able to classify with an accuracy of more than 80% SIRS from the two sepsis groups, based on multiparametric patterns of entropy values in the very low frequencies and indicating reduced metabolic inputs on local thermoregulation, probably associated with extensive vasodilatation.

**Conclusions:**

We suggest that complexity analysis of temperature signals can assess inherent thermoregulatory dynamics during systemic inflammation and has increased discriminating value in patients with infectious versus noninfectious conditions, probably associated with severity of illness.

## Introduction

Fever is a common problem in critically ill patients. While infections are the commonest cause of fever, other noninfectious inflammatory conditions may augment cytokine production with a subsequent febrile or hypothermic response. Investigation of fever involves numerous diagnostic tests that must be performed to differentiate infectious from noninfectious causes and to determine the site of possible infection. Moreover, the presence of fever can significantly increase the cost of treatment in the Intensive Care Unit (ICU) [1,2].

Different biomarkers have been studied for their accuracy in discriminating patients with infectious and noninfectious acute inflammatory states. Many studies have confirmed that procalcitonin (PCT), a 116-amino acid peptide, is induced in the plasma of patients with severe systemic bacterial infections and during severe sepsis and septic shock [3]. However, whereas PCT has been shown to discriminate different causes of fever with an accuracy that exceeds other infection markers (for example, C-reactive protein or leukocyte count), recent systemic overviews and meta-analyses have obtained contradictory results regarding the reliability of PCT in diagnosing sepsis or bacteremia [3-5]. As suggested, the majority of these studies are biased by choice of PCT assay used, severity of illness, focus, and cause of infection, and particularly, by the lack of a reliable gold standard that separates sepsis from noninfectious systemic inflammatory response syndrome (SIRS) [6].

Even though temperature is a continuous quantitative variable, its measurement in the clinical setting has been considered a snapshot of a process, indicating whether a patient is febrile or afebrile (qualitative dichotomous value). However, the complexity of the interactions between different cytokines with pyrogenic and antipyrogenic properties may continuously alter the height and duration of a febrile response. Recently, other diagnostic techniques have been proposed for the association between different properties of the temperature curve with severity of illness in the ICU setting, based on complexity analysis of continuously monitored body temperature [7,8].

We are not aware of any study in the literature investigating a possible association between temperature variability and complexity with different causes and severity of systemic inflammation. Discrimination of inflammatory responses through different patterns of change of temperature-curve complexity would add significant value to such analysis, because various biomarkers have been found to differ in terms of diagnostic reliability in the diagnosis of sepsis [3-5].

In this observational study, we tried to investigate changes of temperature variability and complexity in a mixed population of critically ill patients, during the first 24 hours of an inflammatory state (SIRS) with a suspected infection. In addition, we tried to evaluate through statistical models whether these domains of measurements correlate with causes and severity of systemic inflammation. We supposed that because physiologic rhythms fluctuate over time because of continual interaction between the environment and the internal control mechanisms [9,10], different causes of systemic inflammatory response might alter the dynamic behavior of body temperature.

## Materials and methods

### Setting and studying population

**Figure 1:**
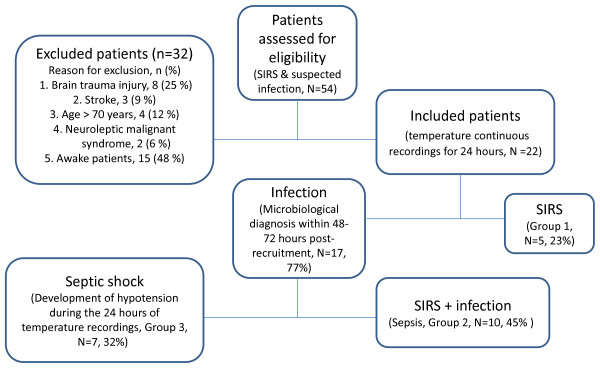
**Flowchart of patients' assessment for eligibility in the study**. Inclusion and exclusion criteria of patients enrolled in the study. SIRS, systemic inflammatory response syndrome.

This study was performed in a mixed eight-bed ICU in the University hospital of Alexandroupolis, Greece, after approval by the local Scientific and Ethics Committee (University Hospital of Alexandroupolis, Institutional Ethics Committee) and after obtaining informed consent from the patient's next of kin. In total, 22 consecutive patients admitted to the ICU from January to September 2011, with a mean Acute Physiology and Chronic Health Evaluation (APACHE) II score on admission 17.4 (± 4.5), were included in the study. The 14 men and eight women had a mean age of 60.86 ± 5.35 years. Every episode of SIRS (an inflammatory state including ≥ 2 of the following: temperature, ≥ 38°C or ≤ 36°C; heart rate, ≥ 90 beats/min; respiratory rate, ≥ 20 breaths/min or PaCO_2 _≤ 32 mm Hg; and white blood cell count, ≥ 12.000/mm^3 ^or ≤ 6.000/mm^3 ^or > 10% immature neutrophils) [11] and suspected infection, such as bacteremia or ventilator-associated pneumonia (VAP) during the patients' stay in the ICU was considered eligible for further analysis (54 patients; Figure 1). Bloodstream infections (BSIs) and VAP were defined according to published guidelines [2]. Patients were excluded from the study (32 patients, Figure 1) if (a) they had neurologic diseases (brain trauma or stroke), due to possible direct or indirect damage of the hypothalamus, probably related to defective thermoregulation [12]; (b) if they had toxic insults, such as neuroleptic malignant syndrome (NMS) associated with administration of antidepressant agents or metabolic crisis, such as thyroid storm; and (3) if they were awake during a suspected infection, because sedation has been found to alter variability measures of different physiologic signals [13]. For that reason, we did not want to study a mixed population of both sedated and awake patients. Categories 1 and 2 are considered to be established noninfectious causes of SIRS that could bias our methods because we were interested only in suspected infectious states. We decided to exclude patients with known metabolic/neurologic causes of admission to the ICU at this stage, which could alter, with different mechanisms, normal thermoregulation. In addition, we decided to exclude patients older than 70 and/or younger than 40 years because of the negative impact of extreme age on complex dynamics of thermoregulatory oscillations [14]. The studied population, 48 to 72 hours after confirming or rejecting the diagnosis of infection based on microbiologic data, was retrospectively divided into three groups: group 1 included patients with SIRS (without proven infection; *n *= 5); group 2, those with sepsis (SIRS + infection; *n *= 10); and group 3, patients in whom severe sepsis (one or more signs of organ failure + infection) and septic shock (hypotension, meaning either systolic blood pressure < 90 mm Hg or mean arterial pressure < 70 mm Hg, despite adequate volume resuscitation; *n *= 7) developed during the period of signal extraction [11]. In addition, a Sequential Organ Failure Assessment (SOFA) score of severity of illness was calculated during the day of temperature recordings, in all patients.

### Temperature measurements

Continuous temperature monitoring was performed during the first 24 hours of the patients' inflammatory response, with a thermistor sensor (Datalogger Spectrum 1000; Veriteq Instruments, Richmond, BC, Canada), attached to the right hypochondrium in all subjects, as was previously described [7,8]. This device is capable of sampling temperature at a rate of 1 sample per 10 seconds (0.1-Hz sampling frequency) with a resolution of 0.05°C. Data were collected daily by using the software *viewLink *and, through Bluetooth technology, were downloaded in separate files for later analysis, to an HP Pavilion 6181, 2 GHz PC, by someone blinded to patients' clinical status.

### Time series analysis

#### Signal preprocessing

**Figure 2:**
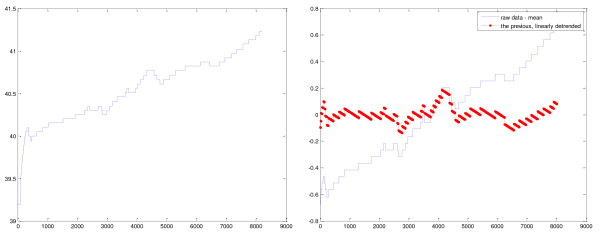
**Preprocessing of temperature signals**. Raw **(a) **and detrended **(b) **temperature curve versus time of recording (minutes) in a patient with ventilator-associated pneumonia and septic shock. The signal average value is subtracted, so that features are not biased by the temperature mean values (1a). Other normalization procedures were also considered, to isolate the long-term trends in the signal (by use of detrend, 1b).

Artifact areas were present in the signals, potentially because of contact and other sensing. These artifact segments were manually annotated and replaced by a linear segment (produced by linear interpolation) connecting the end points of the segment. Then the signal average value was subtracted, so that features were not biased by the temperature mean values, leading to the signal *sign m-*. Further preprocessing was considered (that is, detrending) to isolate the long-term trends in the signal (Figure 2a and 2b). Both versions of the signal (that is, after subtraction of mean value, and the signal *sign_mdetr*, after also estimating and removing linear trend) were studied.

#### Time-scale complexity: wavelet transformation of temperature signals

The temperature signals under consideration are not periodic (in the time frames of interest) or completely random. They seem not to be stationary, in the sense that they contain transients and localized components, whereas their statistics change over time. Time-scale analysis was considered as a method to decompose the signal into a set of subsignals regarded at different scales. Each subsignal contains localized information about the temperature changes in the specific time scale. It is thus possible to examine whether dominant scales are present (also corresponding to frequency content), and what is the dynamic and the patterns of the temperature deviations at different scales (for example, in terms of complexity at different scales).

A wavelet transform is a mathematical tool that can be used to process signals and provide salient information about both the time and frequency content of a transient signal, via the use of a waveform pattern (mother wavelet) of limited duration. Wavelet analysis consists of taking a waveform with an average value of zero, such as the Morlet or Meyer wavelet, and moving it through the extent of the signal [15]. As the waveform is stretched out and scaled, coefficients are produced as a function of both scale and position, representing how well the waveform matches the signal. A wavelet transform can be implemented as discrete wavelets transform (DWT) or continuous wavelets transform (CWT). DWTs use a specific subset of all scale and translation values; whereas CWTs operate over every possible scale and translation values. A wavelet transform has variable time-frequency resolution (that is, good time but poor frequency resolution at high frequencies, and good frequency but poor time resolution at low frequencies) [15,16].

We applied the Meyer mother wavelet for both CWT and DWT. For DWT, based on sampling frequency, nine scales were used, corresponding to the frequency bands depicted in Table 1. Based on previous studies, we assumed that different frequency bands are related to physiologic phenomena, such as (a) neurogenic inputs in association with scale 1 (low frequencies); (b) metabolic inputs related to scales 2 to 3 (very-low frequencies); and (c) unknown influences on ultradian scales, higher than 4 (ultra-low frequencies) [17]. Furthermore, we considered that wavelet features derived from temperature analysis might also reflect microcirculatory fluctuations, related to different local thermoregulatory mechanisms [18,19].

**Table 1:** Scaling of temperature signals with wavelet transform

Scale	Frequency bands (Hz)		Periods of phenomena (minutes)	
	Min	Max	Min	Max
1	0.025	0.05	0.333333	0.666667
2	0.0125	0.025	0.666667	1.333333
3	0.00625	0.0125	1.333333	2.666667
4	0.003125	0.00625	2.666667	5.333333
5	0.001563	0.003125	5.333333	10.666667
6	0.000781	0.001563	10.666667	21.333333
7	0.000391	0.000781	21.333333	42.666667
8	0.000195	0.000391	42.666667	85.333333
9	< 0.0001	0.000195	85.333333	170.666667

Wavelet scales corresponding to different frequency bands of the recording temperature signals, extracted with a sampling frequency of 0.1 Hz, for 24 hours.

For the detail signal of each scale of DWT (that is, the wavelet coefficients in this scale), two features were calculated, the wavelet energy-reflecting variability of the signal and the wavelet entropy, corresponding to the information content within each scale. The wavelet energy (WE) per scale is calculated as follows (Equation 1):

$$WE\left(s_{i}\right)=\overset{L_{i}}{\underset{j=1}{\sum }}w^{2}\left(s_{i},j\right)/L_{i}$$

where *s_i _*is the scale, *s_i _*= 1, 2, ...12. *L_i _*is the total number of wavelet coefficients in scale *s_i_*, and *w(s_i_,j) *is the j^th ^wavelet coefficient in scale *s_i_*.

The wavelet entropy WEn is defined based on the Shannon entropy (Equation 2):

$$WEn\left(s_{i}\right)=-\overset{L_{i}}{\underset{j=1}{\sum }}w_{{}^{n}}^{2}\left(s_{i},j\right)\text{log}\left(w_{n}^{2}\left(s_{i},j\right)\right)\;$$

where the place of probability distributions is taken by normalized squared wavelet coefficients, equivalent to a power-spectrum distribution in a spectral analysis (Equation 3):

$$w_{n}\left({s_{i}}_{,j}\right)={\left|w\left(s_{i},j\right)\right|}^{2}/)\overset{L_{i}}{\underset{j=1}{\sum }}w_{{}^{}}^{2}\left(s_{i},j\right)\;$$

Normalization here is performed per scale (that is, the sum of w_n _per scale is equal to 1). In this manner, after calculating energy and entropy per scale, 18 wavelet features (two features × nine scales) were calculated.

A similar procedure was followed in the CWT, and wavelet energy and entropy were calculated for the neurogenic (CWTen1 and CWTentro1, respectively), metabolic (CWTen2 and CWTentro2), lower metabolic/ultradian band (CWTen3 and CWTentro3) ultradian band (CWTen4 and CWTentro4), as well as for the whole time-scale map (CWTen and CWTentro), leading to 10 CWT features.

Data analysis using wavelets was performed in Matlab (R2006b; The Mathworks, Natick, MA, USA).

#### Complexity features: multiscale entropy (MSE)

MSE was recently introduced for quantifying heart-rate complexity [20]. In brief, for a given time series, multiple "coarse-graining" time series are constructed by averaging the data points within nonoverlapping windows of increasing length τ, where τ represents the scale factor. Subsequently, sample entropy (SampEn) that represents the negative natural logarithm of the conditional probability that two sequences similar for m points remain similar at the next point with a tolerance r [21] is calculated for each time series and then plotted against the scale factor, giving rise to the MSE curve. The sum of SampEn overall scaling factors represents the MSE (sumEn) of a signal. Regular signals are expected to have low sumEn, whereas complex ones take on higher sumEn values. This technique is considered more accurate than approximate entropy (ApEn), which was introduced by Pincus, because of the dependence of ApEn on the record length [22]. For MSE analysis, different values of parameters (m, r, N) are used for calculations. N is the length of the time series. The parameter r, the tolerance for accepting matches, is set between 15% and 25% of the standard deviation (SD) of the time series, after normalization (SD = 1). The parameter m (embedding dimension) is the length of sequences to be compared, and its values vary between 1 and 2 for data length ranging from 100 to 5,000 data points [21,22].

In our analysis, we computed temperature MSE by using 36 scales and after assigning the values of 2 for m and 0.15 for r, by using specific functions in Matlab. For completeness, the basic Sample entropy (without scaling) was also computed.

### Statistical analysis

The aims of this analysis were fourfold:

1. Present descriptive statistics of difference among the groups, revealing the general properties of the temperature signals.

2. Investigate which feature groups form concrete unsupervised clusters, and associate these clusters with the existing medical knowledge.

3. Select features that best classify in a supervised manner, pairwise, SIRS from sepsis or septic shock.

4. Associate temperature features with the clinical picture and severity of the patient.

The methods followed are described in more detail later.

#### No-parametric statistical testing

We applied the Lilliefors test as an adaptation of Kolmogorov-Smirnov test for assessing the null hypothesis that data come from a normally distributed population. None formed the studied variables, except for age, and temperature was found to follow a normal distribution. In this respect, one-way analysis of variance (ANOVA) was used for estimating statistical differences between the three groups of patients for age and temperature.

Nonparametric tests (Wilcoxon rank-sum test) were performed in a pairwise manner for statistically significant differences between (a) SIRS-sepsis, (b) SIRS-septic shock, and (c) sepsis-septic shock for different features derived from variability analysis of temperature signals and for SOFA score. Rank-sum null hypothesis is that data in the two vectors under investigation are independent samples from identical continuous distributions with equal medians.

Furthermore, considering the three groups involved, the Kruskal-Wallis nonparametric test (KW) was applied to assess the significance of the differences in the calculated features among the three groups. The function compares the medians of the samples, testing the null hypothesis that all samples are drawn from the same population (or equivalently, from different populations with the same distribution), as a nonparametric one-way ANOVA and an extension of the Wilcoxon rank-sum test to more than two groups.

Whereas the KW was used to reject the global null hypothesis (that is, whether at least one sample median is significantly different from the others), a further multiple comparison was also used to determine which groups differ significantly in a pairwise manner, with Bonferroni correction for multiple groups.

### Unsupervised clustering

Because we did not know from the beginning in which class (SIRS, sepsis, or septic shock) our patients belonged during data extraction and analysis, we adopted unsupervised learning techniques for cluster generation, based on pairwise distance (dissimilarity) of different wavelet features. In this case, no training set containing known classifications can be evaluated in a test set of different features. So, we calculated pairwise distance contours by using the standard euclidean distance (Equation 4):

$$\text{d}\left(p,q\right)=\text{d}\left(q,p\right)=\sqrt{{\left(q_{1}-p_{1}\right)}^{2}+{\left(q_{2}-p_{2}\right)}^{2}+\cdot \cdot \cdot +{\left(q_{n}-p_{n}\right)}^{2}}=\sqrt{\overset{n}{\underset{i=1}{\sum }}{\left(q_{i}-p_{i}\right)}^{2}}.$$

where d (p,q) is the distance between observations (wavelet features) p and q, and n is the number of elements per observation. This calculation resulted in the distance matrix MDis, which was then visualized with color coding, reflecting the distances and presenting the natural organization of data.

In parallel with the visual inspection, k-means clustering was performed, assigning all the data to two groups, and the unsupervised clustering capability, in terms of intraclass and interclass distance, as well as the relation of these clusters with the *a posteriori *knowledge about the three groups, was quantitatively assessed [23].

We decided to study wavelet entropy and energy per different scales bands for generation of clusters, based on previously published data that demonstrated significant correlations between particular spectral ranges and autonomic or metabolic inputs [17].

#### Feature selection and classification

Although the statistical tests describe the differences among groups and produce features that can potentially lead to the classification of the groups under consideration, it is often the case that multivariate approaches, taking into account combinations and interactions of features, rather than univariate approaches, lead to better results.

In this respect, after confirmation of microbiologic diagnosis and besides statistical testing, a heuristic wrapper method was used to highlight the most informative features, in terms of pairwise classification of temperature signals belonging to the three groups (SIRS, sepsis, and septic shock). The method is based on the repetitive generation of random-feature subsets (here in combinations of four features) from the pool of available features and the evaluation of their classification capacity, in terms of binary linear classification (that is, SIRS versus sepsis, SIRS versus septic shock, sepsis versus septic shock). Successful sets were those reaching classification accuracy higher than a threshold (80%). To address the dataset imbalance present here, the average of sensitivity and specificity was used as a criterion, instead of absolute accuracy. The significance of each feature was measured by an index counting the frequency of appearance of the particular feature in a successful subset. In this respect, the features with the highest significance were selected to form the features sets denoted as *randset *for each one of the three classifiers. The method was implemented as a variation of the *rand features *method available in Matlab.

Subsequently, the selected features sets were used in linear classification with linear discriminant analysis (LDA) [24], and the accuracy was assessed in a leave-one-out cross-validation manner, in which multiple runs took place, and in each run, one sample was presented as the test set, and the remaining samples, as the training set. Because of the small number of data, the results were only indicative of the classification capacity. It must be noted that a multiclass scheme was not considered at this step, as the main focus was the distinction of infection from SIRS, rather than the difference between sepsis and septic shock.

#### Relation with severity and clinical measures

The Spearman rank correlation coefficient (ρ) was used as a nonparametric measure of statistical dependence between different features and a SOFA score of severity of illness. Data are presented as mean ± SD for normally distributed variables or median (25^th ^to 75th percentiles) for measurements without normal distribution. Values of *P *< 0.05 were considered to be significant. Statistical analysis was performed in Matlab (R2006b; the Mathworks).

## Results

Clinical information of all studied patients is demonstrated in Table 2. Mean temperature did not differ significantly between the three groups (38.26 ± 0.26 for group 1, 38.17 ± 1.43 for group 2, and 38.67 ± 0.39 for group 3). However, SOFA scores were significantly different between subjects proved to have SIRS or sepsis versus septic shock [(10 (9 to 10), group 1; and 12 (10.25 to 12), group 2; versus 17 (16.5 to 18), group 3; *P *< 0.001)]. Groups 1 and 2 did not differ in terms of SOFA (*P *= 0.09), whereas age also was found similar among the three classes of patients. All recruited subjects were admitted to the ICU at least 24 hours before development of a suspected infection, except for three patients from group 2 (7, 20, and 21), who were immediately included in the study after being transferred to the ICU from a general ward. Patients from the SIRS group and two patients with sepsis (7,8) were not receiving antimicrobial treatment before developing a systemic inflammatory state. However, the whole studied population was prescribed antibiotics, during the period of temperature recordings, because of a suspected infection. Patients from group 1 and subjects 7 and 8 from group 2 received a combination of β-lactams (carbapenems) and aminoglycosides, whereas the rest from sepsis group and the whole group 3 switched to a new combination of carbapenems, quinolones, and anti-staphylococcus agents. None of our patients received antimycotics during the study period. In addition, all patients in whom severe sepsis and septic shock developed (group 3) received 0.04 to 0.06 μg/Kg/min of vasopressors (noradrenalin) for restoration of adequate blood pressure, according to published guidelines [25]. Moreover, in all septic shock patients and in five subjects from group 2 hyperlactatemia (> 1 m*M*) developed, whereas all patients with pneumonia exhibited arterial hypoxemia (PaO_2_/FiO_2 _< 300) [11]. Low-dose hydrocortisone was not considered necessary at that time.

**Table 2:** Demographic and clinical data of the whole patient population

Case	Diagnosis	T (mean)	T (SD)	SOFA
1	Intestinal ischemia-sepsis	36.27	0.26	11
2	Bacteremia-sepsis	37.55	0.21	9
3	VAP-sepsis	36.47	0.13	12
4	VAP-septic shock	37.17	0.27	16
5	VAP-sepsis	40.56	0.05	10
6	VAP-septic shock	38.25	0.28	18
7	Urosepsis	39.06	0.19	12
8	Hepatic cirrhosis-peritonitis	38.38	0.24	12
9	Polytrauma-SIRS	37.63	0.13	9
10	Pancreatitis-septic shock	38.48	0.18	17
11	Pancreatitis-sepsis	39.46	0.22	13
12	Colectomy-SISRS	38.11	0.38	10
13	Intestinal perforation-septic shock	38.58	0.3	17
14	VAP-septic shock	39.07	0.26	18
15	Colectomy-septic shock	39.22	0.19	13
16	Hepatectomy-sepsis	38.21	0.72	12
17	Abdominal surgery-SIRS	38.63	0.1	8
18	Polytrauma-SIRS	38.78	0.03	12
19	Abdominal surgery-SIRS	38.63	0.1	10
20	Bacteremia-sepsis	39.33	0.17	10
21	Urosepsis	37.23	0.31	12
22	Intestinal ischemia-septic shock	38.87	0.68	18

The 22 patients are presented along with their diagnosis proven within 48 to 72 hours after recruitment in the study, mean value and standard deviation of 24-hour-recording temperature signals, and mean SOFA score of severity of illness during the same day of data extraction. T, temperature; SD, standard deviation; SOFA, sequential organ failure assessment score, during the day of temperature recording; SIRS, systemic inflammatory response syndrome; VAP, ventilator-associated pneumonia.

The statistically significant features, in terms of a nonparametric rank-sum test, when considering the *sign_m *and the *sign_mdetr *temperature signals, are depicted in Table 3, and differences between all patient groups in terms of the KW test are shown in Table 4.

**Table 3:** Distribution of wavelet features in the three groups of patients

Wavelet	Group 1	Group 2	Group 3	1 vs 2	1 vs 3	2 vs 3
Features	(SIRS) *n *= 5	(sepsis) *n *= 10	(septic shock) *n *= 7	*P*	*P*	*P*
***sign_m*, raw data with mean value subtracted**						

WEn(s4)	0.226 (0.19-0.31)	0.159 (0.12-0.21)	0.105 (0.08-0.15)		0.047	
WEn(s5)	0.284 (0.23-0.35)	0.195 (0.12-0.23)	0.144 (0.09-0.16)		0.047	
WEn(s8)	0.485 (0.45-0.53)	0.479 (0.40-0.50)	0.391 (0.36-0.42)		0.010	0.033

***sign_mdetr*, raw data with mean value subtracted and mean trend removed**						

CWTentro	0.040 (0.03-0.05)	0.018 (0.01-0.02)	0.012 (0.011-0.014)	0.04	0.0480	
CWTentro3	0.035 (0.03-0.04)	0.014 (0.009-0.019)	0.009 (0.007-0.011)		0.0480	
CWTentro4	0.029 (0.02-0.03)	0.010 (0.006-0.011)	0.007 (0.006-0.011)		0.0480	
WEn(s4)	0.228 (0.19-0.31)	0.159 (0.12-0.21)	0.105 (0.08-0.15)		0.0480	
WEn(s5)	0.284 (0.23-0.35)	0.195 (0.12-0.23)	0.144 (0.09-0.16)		0.0480	
WEn(s6)	0.389 (0.33-0.41)	0.232 (0.18-0.26)	0.197 (0.18-0.21)		0.0303	
WEn(s8)	0.494 (0.45-0.53)	0.476 (0.39-0.51)	0.383 (0.35-0.42)		0.0101	0.0330
sumEn	4.599 (3.97-5.96)	6.016 (4.62-11.43)	3.683 (2.37-4.60)			0.0136
SampEn	0.007 (0.005-0.009)	0.008 (0.006-0.015)	0.005 (0.004-0.006)			0.0431

The table demonstrates the statistically significant differences of features (*P *refers to rank-sum *P *value), reflecting temperature complexity within different scales and frequency bands, between the three groups of patients. Data are presented as median with intraquartile range (25^th ^to 75th percentiles). CWTentro, entropy derived after continuous wavelet transformation of the whole signal; Wen, wavelet entropy; sumEn, multiscale entropy; SampEn, sample entropy; s, scale.

**Table 4:** Differences between the three groups of patients with respect to the nonparametric Kruskal-Wallis (KW) test and analysis of multiple groups

Features	*P *values
Sign_m	

WEn(s4)	0.0591 (marginally)
WEn(s8)	0.0242

Sign_mdetr	

sumEn	0.0314
WEn(s4)	0.0535
WEn(s5)	0.065 (marginally)
WEn(s6)	0.0592 (marginally)
WEn(s8)	0.0242
CWTentro	0.0446
CWTentro 3 (metabolic)	0.0479

The table shows, for *sign_m *and *sign_mdetr *signal, the statistically significant differences for the three groups, according to the KW test. Wen, wavelet entropy; CWTentro, entropy of the whole signal transformed with continuous wavelet transformation; sumEn, multiscale entropy.

Patients with SIRS exhibited increased wavelet entropy (complexity) in all scales and especially in the ultradian ranges, reflected by wavelet entropy values in scales 3 to 8 of DWT, compared with subjects with sepsis and septic shock, reaching statistical significance between groups 1 and 3. Regarding CWT, the total wavelet entropy exhibited statistical significance among all groups, whereas the entropy in lower metabolic and ultradian scales showed a decrease with sepsis, and statistically significant differences between groups 1 and 3, in accordance with the respective DWT features. These differences were more evident in the analysis of *sign_mdetr *(that is, in the detrended signal). Multiscale entropy (sumEn) and Sample Entropy of the whole signal were decreased mainly in septic-shock patients.

In the KW test, it seems that detrending (*sign_mdetr *signal) increases the number of features with statistically significant differences between SIRS and sepsis, whereas these differences are potentially masked in the signal when only the mean value is removed (*sign_m*), because of the overall signal trend, or because of the boundary effects.

**Figure 3:**
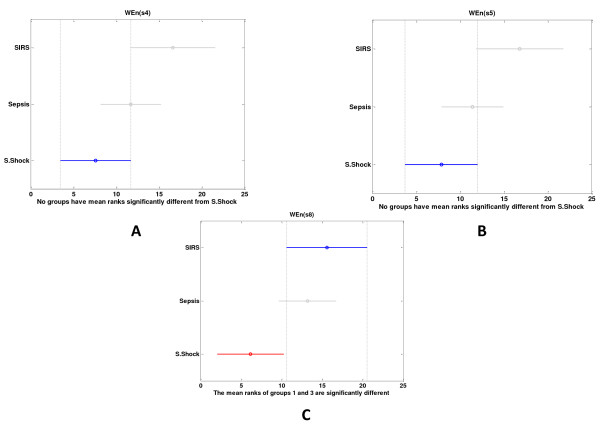
**(a, b) Multiple comparisons of different studying groups for *sign_m***. The visual representation of the multiple comparisons windows groups with Bonferroni correction, Kruskal-Wallis (KW) mean ranks, and confidence intervals depicted here, for *sign_m*. The three groups differ in terms of WEn (s4, s5, s8), but only the last feature reaches statistical significance between patients with SIRS versus septic shock.

**Figure 4:**
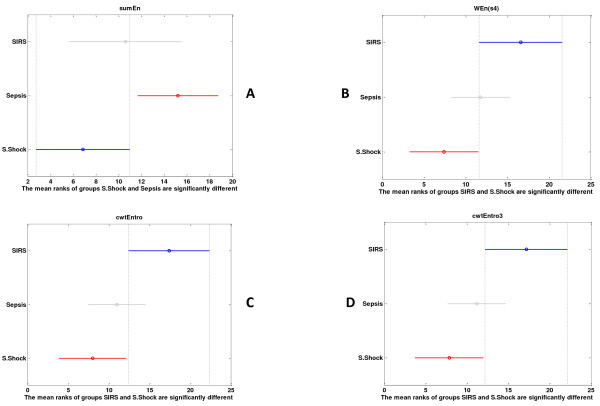
**(a through d) Multiple comparisons of different studying groups for *sign_mdetr***. KW mean ranks and confidence intervals depicted here, for *sign_mdetr*. Multiscale entropy (sumEn) of the whole signal, continuous wavelet entropy (cwtEnrto) for the whole signal and for scale 3, and finally, wavelet entropy per scale 4 (WEn(s4)) exhibited significant differences between different groups of patients.

The multiple comparisons with Bonferonni correction for the raw signal (*sign_m*) showed that wavelet entropy in the ultradian bands was more increased in SIRS than in sepsis and reached statistical significance in septic shock (Figure 3). This is more obvious and is observed in more features, including CWT features, for the detrended signal (*sign_mdetr*) (Figure 4).

**Figure 5:**
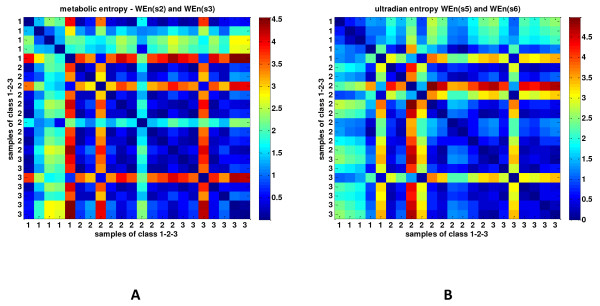
**Clusters of dissimilarity between the three groups of patients**. Pairwise distance and visualization of the dissimilarity matrix, based on the euclidean distance, between wavelet entropy of scales reflecting metabolic **(a) **and other unknown inputs **(b) **on very-low-frequency (scales 2 to 3, 0.025 to 0.006 Hz) and ultra-low-frequency bands (scales 5 to 6, 0.003 to 0.0007 Hz), respectively. Red color reflects dissimilarity (high distance), and blue color, similarity (small distance) between patients belonging to groups 1, 2, and 3.

Figure 5 (a and b) demonstrates the clustering of the whole patient population according to the pairwise euclidean distance of metabolic (WEn (s2 and 3)) and ultradian entropy (WEn (s5 and 6), illustrated in a dissimilarity matrix, with deep blue denoting small distance (that is, similarity), and deep red denoting big distance (that is, dissimilarity). It seems that patients with sepsis and septic shock exhibited similar patterns of complexity in these scales (their distance forms in deep blue areas in the figure), whereas subjects with SIRS form a rather distinct area of similarity, and in most cases, the distances between cases belonging to SIRS and sepsis are higher.

The outcome of the visual inspection was further reinforced by the quantitative clustering results depicted in Table 5, for the *sign_mdetr *temperature signal. The quantitative clustering measures suggest that the most concrete clusters are formed with the DWT entropies of the ultradian band, as well as with the CWT entropies. These clusters have also a direct correspondence with the SIRS or sepsis/shock classes. Conversely, clusters based on temperature mean value and standard deviation, or on wavelet energies are neither well formed, nor do they correspond to the groups in focus. Clusters formed by sample entropy and sumEn seem concrete but do not correspond well to the three groups investigated in this work.

**Table 5:** Clustering measures

	Cluster points		Intraclass distance		Interclass distance					
	**n1**	**n2**	**c1**	**c2**	**d12**	**d21**	**Clustering cost**	**Sensitivity**	**Specificity**	**Accuracy**

DWT high ultradian entropy (WEn5-6)	15	7	8.73	6.73	3.85	3.86	1.673	82.35%	80%	81.81%

CWT entropy neuro meta	5	17	6.15	6.79	4.25	4.24	1.749	60%	88.23%	81.81%

CWT entropies	8	14	10.61	4.11	3.61	2.79	1.777	100%	82.35%	86.36%

CWT entro ultradian	14	8	3.95	10.76	2.68	3.57	1.788	82.35%	100%	86.36%

SampEn and sumEn	17	5	8.43	6.34	3.28	3.83	1.905	76.47%	20%	63.63%

CWT entro all and neurogenic	5	17	6.78	8.73	4.21	4.09	1.990	60%	88.23%	81.81%

DWT low ultradian entropy (WEn7-8)	15	7	13.49	9.46	3.17	3.44	2.402	76.47%	60%	72.72%

DWT Neurogenic and metabolic entropy (WEn1-2-3)	6	16	9.63	11.71	5.83	6.36	2.420	60%	82.35%	77.27%

T Mean & Std	9	13	11.63	12.78	2.35	2.13	2.499	40%	58.82%	54.54%

CWT energy	4	18	14.48	11.92	8.17	6.35	4.353	0	76.47%	59.09%

The table shows, for *sign_mdetr *signal, clusters formed for different feature sets, number of data per cluster (n1 and n2), along with the intraclass euclidean distance for each cluster (c1 and c2), the mean interclass distance to center of the class (d12 and d21), the cost, calculated as the sum of intraclass and inverse interclass distances, and the correspondence of clustering with the *a posteriori *knowledge of groups (as accuracy, sensitivity, and specificity, with sensitivity referring to SIRS). The schemes are sorted in terms of cost. Schemes resulting in clustering with fewer than three members are omitted. DWT, discrete wavelet transformation; CWT, continuous wavelet transformation; SampEn, sample entropy; sumEn, multiscale entropy; SampEn, sample entropy; T, temperature; Std, standard deviation.

Furthermore, weak but statistically significant anticorrelations were observed between SOFA score and WEn (6), WEn (8), sumEn, and CWT energy (ρ = -0.461, -0.605, -0.499, and -0.564; *P *< 0.05 for all comparisons, respectively) in the whole group of patients for sign_mdetr, whereas for sign_m, the most significant anticorrelations were found between WEn(s8), sumEn, and SOFA (ρ = -0.605 and -0.563, respectively; *P *< 0.05). These results imply that high wavelet entropy in ultra-low frequencies and multiscale entropy might be related with a less critical case.

Table 6 depicts the outcome of the feature selection and linear classification. The different randset schemes of selected features with the best classification accuracy between patients with SIRS versus sepsis (or septic shock) was found to include DWT wavelet entropy (WEn) and energy (WE) at scales 5, 6, and 8 (ultradian frequencies) or CWT wavelet entropy in ultradian frequencies (CWTentro4), succeeding in all cases with more than 80% accuracy, in a leave-one-out cross-validation manner.

**Table 6:** The pairwise classification results

Groups		Feature set	Accuracy	Sensitivity	Specificity
**SIRS vs Sepsis**	*sign_m*	WE(s2)	40%	80%	20%

	*sign_m*	WE(s2), WEn(s6)	80%	80%	80%

	*sign_mdetr*	CWTentro4	80%	80%	80%

	*sign_mdetr*	CWTentro4, WE(s5), CWTene	93.33%	100%	90%

**SIRS vs S. Shock**	*sign_m*	WEn(s5)	83.33%	80%	85.71%

	*sign_m*	WEn(s5), WEn(s6), WEn(s8), WE(s8)	91.67%	80%	100%

	*sign_mdetr*	WEn(s6)	83.33%	80%	85.71%

	*sign_mdetr*	WEn(s6), CWTentro4, WE(s8)	100%	100%	100%

The table demonstrates the *randset *feature sets and the classification performance achieved with a linear classifier and leave-one-out cross-validation, in terms of accuracy, sensitivity, and specificity. Here, sensitivity refers to SIRS, and specificity refers to sepsis or septic shock, respectively. The results are presented for *sign_m *and *sign_mdetr*, separately. Both univariate models for the best feature selected, and multivariate models, are depicted. WE, wavelet energy; Wen, wavelet entropy; CWT, continuous wavelet transformation; s, scale; entro4, entropy per scale 4.

**Figure 6:**
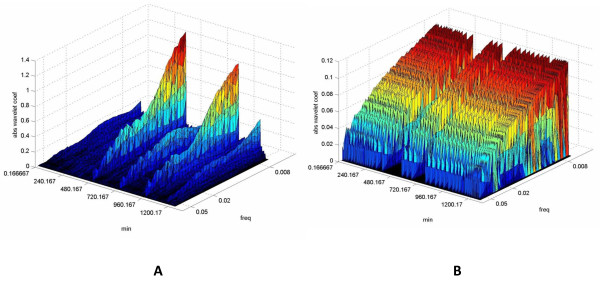
**Continuous wavelet transformation (CWT) of temperature curves in two patients with sepsis and septic shock**. The figure depicts two examples of CWT of temperature recordings from a patient with VAP and sepsis **(a) **and from someone with pancreatitis and septic shock **(b)**. The three-dimensional plots include absolute wavelet coefficients, time (minutes), and frequency (Hz). The third dimension (amplitude) that corresponds to the wavelet coefficient is represented by a color palette in which blue-violet is the minimal value, and purple-red is the maximal value. The wavelet coefficients are relative to the signal under study and provide information regarding its variability within a frequency band (scale). Coefficients in very-low and ultra-low-frequency ranges in case **(a) **seem increased in relation to case **(b)**. Moreover, an apparent irregularity of coefficients' distribution exists in the first compared with the second graph, indicating reduced complexity and reduced amount of inputs on temperature regulation within very-low and ultra-low frequencies in the patient with septic shock.

Figure 6 (a and b) depicts two examples of CWT from a patient with pneumonia and sepsis and from someone with septic shock and pancreatitis, respectively.

## Discussion

In our study, complexity metrics within low-, very-low-, and ultra-low-frequency bands were proven to discriminate successfully between patients with SIRS, sepsis, and septic shock.

Because of the small data set and to show in the best way the virtue of the proposed features, an extended analysis took place. For this reason, both univariate and multivariate linear classification was performed, showing that some of the univariate models are already quite satisfactory, with an improvement by multivariate analysis. Furthermore, the value of the proposed features was reinforced by the use of clustering techniques, in which quite concrete clusters were formed, related to the groups under investigation.

Different studies showed that the low-amplitude fluctuations of skin temperature are caused by rhythmic alterations in peripheral blood flow, linked with oscillations of smooth muscle tone. Particularly, only low- and very-low-frequency fluctuations of simultaneously recorded blood flow and temperature measurements seem to be correlated significantly because of the exponential decay of the temperature amplitude, in relation with the spectral content of the signal [18,19]. For these reasons, correlations within the frequency range from 0.14 to 2 Hz are compatible with the values of noise correlations. In a study of Shusterman and Barnea [26], it was shown that skin-temperature fluctuations between 0.01 and 0.03 Hz were significantly reduced in response to different types of stress. These oscillations were attributed to sympathetic nervous system activity, related to both local autoregulation and reflex neurohumoral control of blood flow.

Different authors using a wavelet-based technique for assessing synchronization and coupling between peripheral skin temperature and blood-flow signals, found increased coherence higher than for uncorrelated white noise, in two frequency intervals, around 0.1 Hz and 0.007 Hz [18,19]. Although oscillations at approximately 0.1 Hz were attributed to myogenic activity, the latter spectral range was proven to correlate with biochemical processes at the level of endothelium [17-19]. The endothelium plays a pivotal role in regulating blood flow, controlling the contraction and relaxation of smooth muscle cells by a release of different vasodilators, such as NO or vasoconstrictors [27]. The production of these molecules can be altered during a systemic inflammatory response to different noxious stimuli [28].

In our study, low- and very-low-frequency components of temperature curves exhibited decreased variability and complexity in patients proven to have sepsis and shock, compared with SIRS. Maybe this could be attributed to reduced local blood flow in the first two groups, which seems to reduce amplitude of vasomotion [29], related with blood-flow redistribution during severe inflammation. Although a possible pathophysiologic link remains unknown, the reduction in metabolic inputs with local thermoregulation could reflect changes in the dynamics of the release of different molecules from the endothelium. For instance, highly and continuously expressed inducible NO synthase (iNOS) in endothelial cells with subsequent increased levels of NO in patients with severe sepsis and septic shock could be associated with reduced smooth muscle cell oscillations. Moreover, cytopathic hypoxia, reduced microcirculatory flow, or number and interaction of vascular territories with shunt hypoxia that seem to occur during severe sepsis and septic shock could also be related with low local metabolic activity [28]. Concerning ultra-low-frequency variations, alterations of different rhythmic processes, such as oscillations between rapid-eye movement (REM) and non-REM sleep, pituitary hormonal secretions or NF-κB cellular signaling pathways, have been supposed to influence ultradian rhythms in both humans and animals; however, their possible effects on skin-temperature oscillations during systemic inflammation remain unknown [30-32].

Except for considering fluctuations of local cutaneous circulation as a possible cause of alterations in temperature oscillatory phenomena during SIRS or sepsis, other centrally acting mechanisms cannot be excluded. For instance, circadian rhythms of tumor-necrosis factor-α (TNF-α) receptors have been linked with central thermoregulation, especially during immune activation from endotoxin, by modulating the availability of free TNF-α [33]. A possible association between oscillatory behavior of TNF-α and rhythmic neuronal NF-κB activity, which has been found to affect thermoregulation [34], could be related to changes in ultradian spectral fluctuations of temperature signals during infection.

In our study, we decided to exclude many patients eligible for further analysis based on our inclusion criteria, aiming to increase homogeneity as much as possible. For that reason, all studied individuals were sedated during temperature recordings, something that enhances accuracy of our results, because anesthesia has been found to affect negatively the amplitude of different frequency components of microcirculatory flow, estimated with wavelets [35]. In addition, patients with already proven neurologic (11 patients), metabolic, or other toxic causes of SIRS (two patients) were not included, because we were interested only in cases of suspected infection according to published guidelines [2]. Patients did not differ in terms of APACHE II score on admission; however, subjects with septic shock had a higher SOFA score on the day before development of a suspected infection and inclusion into the study (data not shown). For these reasons, previous immunologic status cannot be excluded as a possible confounder to our findings.

In this study, we cannot exclude misdiagnosis of some patients with infection not proven by the microbiologic laboratory, because no gold standard exists for the separation of SIRS from sepsis, and everybody was receiving antimicrobial treatment during recruitment. Nonetheless, we believe that the *a posteriori *classification of patients in the three groups, after confirmation of infection, seems more or less accurate, because none of patients with SIRS was receiving antibiotics before inclusion in the study, whereas all diagnoses of infections were based on recently published guidelines [2] and on positive results from quantitative cultures, approximately 72 hours after development of a systemic inflammatory state.

The small number of patients is a significant limitation; however, the adoption of multivariate tools with different data-mining techniques led to a classification with an accuracy of 80%. To avoid possible overfitting of data, we tried to implement both univariate and multivariate models and included only two features in our clustering schemes, whereas a cross-validation technique also was adopted. However, validation with a more-extended data set is a necessary future step.

The adoption of such methods for monitoring of different physiologic parameters fulfills the requirements of contemporary critical care medicine for better and more-accurate early warning signs for patients, because they are based on high-frequency measurements and are much easier to get at the bedside. In addition, variability analysis reveals information that is "hidden" with conventional monitoring techniques. For that reason, in our study, we were not interested in mean temperature values, which are not representative of true core temperature and can also vary between different places of measurement, but we wanted to track dynamic changes of continuously monitored temperature, indicative of a dysegulated homeostasis of a complex thermoregulatory system during severe inflammation [7,8]. These results of reduced temperature complexity during severe sepsis and septic shock are in line with findings from other studies, showing that critical illness alters the inherent dynamics of different physiologic signals, indicative of a system "decomplexification" [9,36]. Nonetheless, an assessment of the potential impact of different places of measurement on temperature variability could be the aim of a future study. Subsequently, we suggest that a wavelet-based classification rule could guide clinicians, 24 hours after a suspected infectious episode, to decide properly about their patients. However, validation of our preliminary results in a larger and more heterogeneous cohort of patients will strengthen our findings.

Furthermore, a comparison between a "physiomarker" and a biomarker model, including different biomarkers such as PCT, could add significant value to our results. Nonetheless, standardization of different biosignal-processing techniques, appropriate selection of different parameters, sampling frequency, or noise filtering is urgently needed.

Finally, the development of a real-time system of risk stratification for clinical deterioration due to infection and using continuous temperature monitoring could provide early markers of sepsis or development of septic shock. Such efforts have already managed to track changes in variability and complexity of heart-rate signals in sepsis patients, much earlier than an increase in body temperature [37,38].

## Conclusions

A healthy state exhibits some degree of stochastic variability and complexity in physiologic variables, such as temperature. This variability accompanies healthy systems and has been suggested to be responsible for their greater adaptability and functionality related to pathologic systems [39]. Critical illness seems to disrupt normal rhythms, giving rise to more periodic patterns in a system's output, such as temperature. Different techniques have been used for assessing complexity of inherent dynamics of physiologic signals. Wavelet transformation seems to have many advantages over other time-series processing techniques, because it can assess both variability and complexity of temperature oscillations in different frequency bands that have been found to be affected by both neurogenic and endothelial influences. The early and accurate discrimination of a systemic inflammation, based on the presence or absence of infection, is a difficult task, and the adoption of such quantitative methods could add significant value to the already existing biomarkers, because they are inexpensive, noninvasive, and permit continuous monitoring of patient status. We suggest that temperature complexity in very-low and ultra-low frequencies is able to classify patients with SIRS, sepsis, and septic shock, possibly reflecting severity of illness. However, because of the small sample size, these findings remain indicative, and larger groups must be studied for validating the diagnostic accuracy of our methods.

## Key messages

• Analysis of continuously monitored temperature signals in critically ill patients with different processing techniques can be of significant value for the discrimination of patients with infectious or noninfectious causes of inflammatory states.

• The use of wavelet analysis seems to offer a significant benefit in relation to other tools, because it can detect changes of both variability and complexity in different frequency bands of temperature signals, which have been shown by others to correlate with physiologic phenomena.

• An increased complexity in all spectral ranges of temperature curves, and particularly in ultradian frequencies, is observed in patients with SIRS, versus sepsis and septic shock.

• The temperature oscillations in the region of very-low and ultra-low frequencies that are influenced by endothelial and other unknown inputs, respectively, are significantly reduced in patients with sepsis and septic shock versus SIRS.

## Abbreviations

APACHE: Acute Physiology and Chronic Health Evaluation; ApEn: approximate entropy; BSI: bloodstream infection; CWT: continuous wavelet transform; DWT: discrete wavelet transform; ICU: intensive care unit; KW: Kruskal-Wallis; LDA: linear discriminant analysis; MSE: multiscale entropy; NO: nitric oxide; PCT: procalcitonin; REM: rapid eye movement; SampEn: sample entropy; SIRS: systemic inflammatory response syndrome; SOFA: sequential organ-failure assessment score; sumEn: sum of sample entropies in different scales during MSE analysis; TNF: tumor necrosis factor; WEn: wavelet entropy; Wes: wavelet energy per specific scale(s).

## Competing interests

The authors declare that they have no competing interests.

## Authors' contributions

All authors read and approved the final manuscript. VEP was the principal investigator who designed the study, collected data, helped with data analysis, and wrote the manuscript. IGC was responsible for data analysis, NKM reviewed, edited, and finally approved methods of data analysis. IAP supervised the whole study.
